# The impact of resilience as a protective factor on Health-Related Quality of Life’s psychological dimensions among adolescents who experience peer victimization

**DOI:** 10.1038/s41598-022-23424-1

**Published:** 2022-11-07

**Authors:** Ángela de Lourdes Martín-Pérez, Inés Morán-Sánchez, Juan José Gascón-Cánovas

**Affiliations:** 1 Department of Psychiatry, Santa Lucía Hospital, Cartagena, Spain; 2 Health Service of Murcia, Cartagena Mental Health Centre, Murcia, Spain; 3 IMIB-Arrixaca, Murcia, Spain; 4 Department of Preventive Medicine and Public Health, Faculty of Medicine, University of Murcia, Campus de Espinardo, 30110 Murcia, Spain

**Keywords:** Psychology, Environmental social sciences, Medical research

## Abstract

Peer victimization have a negative impact on Health-Related Quality of Life (HRQoL) during adolescence, however some personal skills such a person's resilience could play a significant role in this relationship. In this context, this study aims to analyse if resilience is a moderator of the relation between peer victimization and HRQoL’s psychological dimensions. Sociodemographic data, peer victimization, psychological domains of HRQoL and resilience were measures in a sample of 1428 secondary school students using the following scales: “Adolescent Peer Relations Instrument-Bullying”, “KIDSCREEN-52” and “Brief Resilient Coping Scale. Different multivariate analyses were carried out using linear regression. PROCESS tool was used to examine the moderating role of resilience, with John-Neyman post-hoc approach to quantify moderation. Results suggest that resilience could moderate the association between physical (β =  − 0.0021; *p* = 0.025) and verbal victimization (β =  − 0.0018; *p* = 0.024) and the “Mood and Emotions” dimension of adolescents’ HRQoL. Nevertheless, this regulating influence appears to be faint (∆R2 0.004). Resilience showed no moderating effect between social victimization and psychological dimensions of HRQoL. We concluded that resilience could function as a protective factor that weakly regulates the negative association between physical and verbal victimization and the psychological sphere of adolescents’ HRQoL.

## Introduction

Peer victimization has been suggested as a type of abuse in which an adolescent or child is frequently the object of aggressive and/or unsolicited behaviour (victim) from one or more schoolmates (bullies)^1^. It is a societal phenomenon that has become increasingly common and problematic^2^, representing the most prevalent type of violence during school years^3^, especially in adolescence^4^. According to World Health Organization^5^, one-third of children and teenagers have been bullied by their peers at least once in their lifetime. Specifically, in Spain, the reported lifetime prevalence of peer victimization is even higher^6^ with a probability of having been harassed by colleagues ranging from 48.8% to 62.2%^7,8^.

Negative actions within traditional peer victimization can be physical (e.g. punching, pushing, kicking, hitting), verbal (e.g. teasing, threatening, name calling) and social (e.g. rumour spreading, social exclusion)^9^. In addition, with the incorporation of new technologies into our digital society, recent forms of attack on the Internet– cyberbullying – have recently emerged^10^, with an increasing time trend^11^.

The impact of peer victimization on adolescents’ health has been extensively studied during the past decades^12,13^. This period of life is especially susceptible to victimization effects due to the importance of social relationships, the intensity in the perception of emotions and biological changes ^14^. Researchers have documented how experiencing victimization by peers, in any of its forms, affects negatively youths’ physical and mental health^15–17^. Health-Related Quality of Life (HRQoL), a multidimensional concept that covers functional status and individual assessment of physical, mental and social health ^18^, is one of the health correlates related to peer victimization. It is well-recognized that adolescents who are victimized by their peers, and especially those who experience more than one form of victimization ^19^, have a lower HRQoL compared to youths who are not involved^20–22^. Moreover, recent research has shown that all types of peer victimization negatively affect HRQoL and, in particular, its psychological domains. Of all forms of peer victimization, physical violence appears to be the one that most negatively affects adolescents’ HRQoL^23^. However, victims can present different psychological and emotional outcomes even if they faced the same type of harassment.

Little data is still known about protective factors that might facilitate better psycho-emotional HRQoL among teens who have suffered from peer victimization. Protective factors are defined as elements that can mitigate the harmful impact of being exposed to risk, in this case, victimization by peers^24^. Resilience has been emphasized as a widely studied protective factor by several authors^25–27^. It has been conceived as a personal trait, a result or a process of adaptation^28^ that leads a person to recover from negative emotional experiences or, ultimately, adversity ^29^. Previous studies have analysed how resilience mediated the relation between peer victimization and negative outcomes on HRQoL ^20^, subjective well-being ^30,31^ or youth’s mental health problems^27,32,33^. All the studies previously mentioned show that resilience plays a role in regulating the harmful impact that peer victimization has on different health’s correlates. However, the extent to which it is able to modulate this impact is controversial. Moreover, little is known about the association between different types of peer victimization, resilience and its effect on specific dimensions of HRQoL.

Accordingly, this study was conducted to examine the association between psychological domains of HRQoL and resilience among adolescents exposed to different forms of peer victimization (physical, verbal and social). Consequently, we analysed if resilience is a moderator of the relation between peer victimization and the “psychological well-being” and “mood and emotions” dimensions of the HRQoL. As a secondary objective, it was also studied whether there were differences in adolescents’ resilience levels according to sociodemographic factors.

## Methods

### General design and participants

A retrospective cross-sectional study was conducted including secondary school students (12–16 years old) recruited from all academic centres in a city located in south-eastern Spain (*n* = 1476). Every adolescent meeting the inclusion criteria was invite to take part in the present research. The inclusion criteria were: (a) being a secondary school student, (b) fluency Spanish to understand the questionnaire, (c) having obtained legal tutors’ written consent before joining the study.

Before starting the fieldwork, a letter was sent to the city council’s Department of Education to inform them about the research project and approval was obtained. Department of Education transmitted the information to the participating secondary schools’ heads and, they, to major teachers of each class. Several meetings between the research committee and secondary schools’ heads took place to discuss the protocol to be followed as well as to plan the logistics and data collection methods.

Information was obtained during the last 30 days of the school year from self-completed questionnaires administered by major teachers during class time. Data collection was supervised by qualified staff of the research committee. Adolescents were given one hour to complete the entire questionnaire. All questionnaires were completed anonymously by students. Written consent was obtained from parents via Parents’ Associations in all participating schools before joining the study.

The present study was approved by the Research Ethics Committee of Murcia’s University.

### Instruments

Sociodemographic variables were collected by means of a questionnaire designed ad hoc. The variables included were gender, age, family structure (*Nuclear, Mononuclear or No parents at home*), ethnic origin (parents’ birthplace: *both Spanish*, *one Spanish*, *Maghreb*, *Latin-Ecuador* or *Others* which included all other options) and parental educational attainment. Based on the procedure proposed by the Spanish Society of Epidemiology^34^, information about social class in terms of parents’ employment was also included. For both parental educational attainment and social class, the highest positions of both parents were taken as a reference.

Peer victimization was measured using the validated Spanish version of the “Adolescent Peer Relation Instrument-Bullying (APRI)”, developed by Parada^35,36^. This scale comprises 18 items and measures three different dimensions of peer victimization: physical (6 items), verbal (6 items) and social (6 items). Each item is rated on a 4-point Likert scale *(0* = *Never/seldom, 1* = *Frequently, 2* = *Very often, 3* = *Constantly)*, which indicates the frequency of peer victimization suffered by an adolescent from the beginning to the end of the academic year (9-month retrospective follow-up)*.* The score for each dimension was calculated as the sum of the respective items. The higher the score on each subscale, the stronger the victimization suffered by the teenager.

Adolescents were considered victims of each form of victimization if they reported having suffered “frequently” at least one of the behaviours indicated in the questionnaire during the last academic year.

HRQoL was measured by analysing two out of ten dimensions of the validated and adapted to Spanish version of the KIDSCREEN-52 questionnaire^37^: *Psychological Well-being* (6 items) and *Mood and Emotions* (7 items). Each item is rated on a five-point Likert scale corresponding to feelings of well-being over the previous week *(Never* = 1, *Seldom* = 2, *Sometimes* = 3*, Often* = 4, or *Always* = 5*).* Scores are calculated independently for each dimension as T- values of the Rasch scores corresponding to the sum of the response options ^38^. The higher the score on each dimension, the higher the quality of life related to that dimension.

Resilience was assessed using the validated and adapted to Spanish version of the “Brief Resilient Coping Scale (BRCS)”^39,40^. This scale comprises 4 items and measures a person’s ability to cope with stress in a highly adaptative way. Each item is rated on a five-point Likert scale corresponding to the degree of agreement that the person feels about him/herself, ranging from “*1* = *completely disagree*” to “*5* = *completely agree*”. The score for resilience was calculated as the sum of the four items, with higher scores denoting higher resilience levels. Resilience total scores were standardised on a scale from 0 to 100 points in order to ease its interpretation.

### Statistical analysis

All descriptive and inferential analyses were performed using the Statistical Package for Social Sciences SPSS – 24.0 and the statistical package Stata, version 16.0. *p*-values < 0.05 were considered to be statistically significant.

The adolescents’ sociodemographic characteristics were analysed using descriptive analysis calculating frequencies and percentages. The mean scores and standard deviations (SD) of the scale measuring adolescents’ resilience levels were calculated according to sociodemographic characteristics. Any sociodemographic related differences were tested by applying a one-way analysis of variance (ANOVA)^41^. Welch’s t-test was applied to test the hypothesis of means’ equality between the different categories of the studied variables^42^. Differences between groups were assessed using Tamhane’s T2^43^ when variances were heterogeneous while Fisher’s Least Significant Difference (LSD) test^44^ was applied when variances were homogeneous.

Several multivariable linear regressions^45^ were carried out to study the associations between peer victimization and resilience on psycho-emotional dimensions of HRQoL. To detect whether resilience moderated the association of peer victimization on adolescents’ HRQoL, Hayes PROCESS tool was used to examine the moderating role of resilience ^46^. To explore moderating effects, post hoc analyses were conducted using the Johnson–Neyman technique with Hayes’s PROCESS. Before carrying out linear regression analyses, assumptions associated with a linear regression model were checked for compliance^47^. Linearity and homoscedasticity were checked by using scatter plots. Normality was tested by P-P plots. Independence was verified by calculating Durbin-Watson statistics and by checking their values were between 1.5 and 2.5. The absence of multicollinearity was tested by measuring Tolerance (> 0.10) and Variance Inflation Factor (< 5). Each form of peer victimization (physical, social and verbal) was taken as an independent variable. On the other hand, “Psychological well-being” and “Moods and Emotions” were taken as dependent variables. Resilience was introduced as the moderating variable. Gender and age were also inputted as covariates. Gender was handled as a dummy variable, considering as reference category “girl” (girl = 0; boy = 1). Analyses were carried out by calculating β coefficient at a 95% Confidence Interval (CI).

A series of confirmatory factor analysis^48^ by means of the corresponding Structural Equation Model were used to determine the validity of the scores of the three scales used according the theoretical internal structure of each questionnaire (a first order model consisting of three and two correlated factors for the APRI and KIDSCREEN respectively and a one-dimensional first order model for the BRCS). Therefore, Maximum Likelihood method with Satorra Bentler (SB) correction for non-normality^49^ was used to estimate parameter and calculated goodness-of-fit indices. In this sense, we estimate the goodness of fit of the scales using the “Root Mean Square Error of Approximation” (RMSEA), the “Comparative Fit Index” (CFI), the Tucker-Lewis index (TLI) and the “Standardized Root Mean Square Residual” (SRMR) using the Satorra-Bentler scaled chi^2^ statistic in their calculation. RMSEA < 0.05, CFI and TLI > 0.95 and SRMR < 0.05 indicate good fit for model while RMSEA < 0.08, CFI and TLI > 0.90 and SRMR < 0.06 indicate a reasonable fit^50^. In addition, stability of measurements or consistency of each subscale was measured using the “composite reliability” (CR) coefficient and taking into account the values of the “standardized factors loadings for the indicators” and the “variances of the error for the indicators” obtained in the corresponding CFA^51^. CR values above 0.7 were found satisfactory^52^.

### Ethics approval

The present study was approved by the Research Ethics Committee of Murcia’s University. All procedures performed in the study were in accordance with the ethical standards of the national research committee and with the 1964 Helsinki declaration and its later amendments or comparable ethical standards.

### Consent to participate

Informed consent was obtained from all individual participants (and their parents/legal guardians) included in the study.

## Results

### Demographic characteristics and prevalence of peer victimization among adolescents

**Figure 1:**
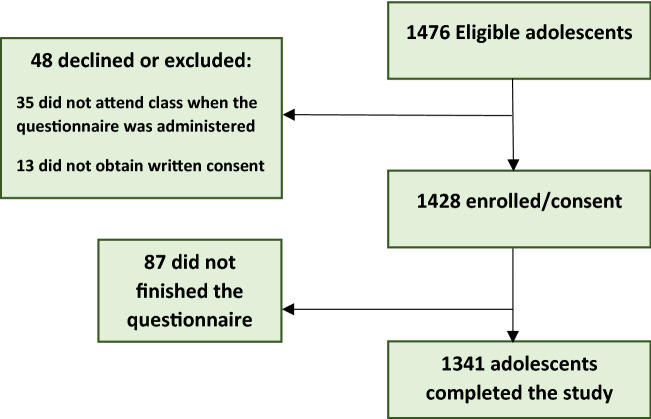
Flow chart of the study population.

Thirty-five adolescents of the eligible population did not obtain written consent from their legal tutors and 13 teenagers did not obtain written consent. From the remaining students, 1341 of them finally completed the entire questionnaire (effective participation rate = 90.8%). Figure 1 shows the participant flow chart during the study.

Participants included 709 boys (52.9%) with a mean age of 14.6 years (SD = 1.2) and an age range of 12 – 18 years. The majority (85.6%) belonged to a nuclear family, in which both parents were Spanish (64.7%). Two–thirds of the main breadwinners worked in semi-skilled or unskilled manual jobs, and less than one fifth had higher education.

One quarter of the participants reported having been victims of at least one form of victimization. (Table 1).

**Table 1:** Demographic characteristics, resilience and prevalence of peer victimization among adolescents (n = 1341).

	n	(%)
**Gender**		
Female	632	47.1
Male	709	52.9
**Age range (years)**		
[12.0–13.9]	490	36.5
[14.0–15.9]	651	48.5
[16.0–18.9]	200	14.9
**Type of family**		
Nuclear	1148	85.6
Mononuclear	178	13.3
No parents at home	15	1.1
**Parental ethnic origin**		
Both Spanish	867	64.7
One Spanish	38	2.8
Maghreb	243	18.1
Latin-Ecuador	131	9.8
Other	62	4.6
**Social class**^**a**^		
I-II	272	20.3
III	131	9.8
IV-V	832	62.0
VI	106	7.9
**Parental educational attainment**		
No education/primary education	435	32.4
Secondary education	626	46.7
Higher education	280	20.9
**Physical victimization**	129	9.6
**Social victimization**	212	15.8
**Verbal victimization**	241	18.0
**Any form of victimization**	334	24.9
**Resilience, mean (SD)**	61.4 (25.3)	

*Population’s total number (n) and percentages (%) according to sociodemographic characteristics.*

*SD: Standard deviation.*

a. Social class: I. Higher managerial; II. Intermediate managerial; III. Supervisory and junior managerial; IV. Skilled manual occupations; V. Unskilled manual occupations; VI. Unemployed/pensioner/retiree.

### Validity and reliability of the scores of the scales in the study sample

Confirmatory Factor Analysis (CFA) showed a reasonable fit to the sample data for the APRI (95%CI RMSEA = 0.062–0.071; RMSEA(SB) = 0.027; CFI(SB) = 0.964; TLI(SB) = 0.958; SRMR = 0.035), the two KIDSCREEN domains (95%CI RMSEA = 0.070–0.082; RMSEA(SB) = 0.066; CFI(SB) = 0.933; TLI(SB) = 0.918; SRMR = 0.043), and the BRCS questionnaire (95%CI RMSEA = 0.001–0.083; RMSEA(SB) = 0.009; CFI(SB) = 0.999; TLI(SB) = 0.999; SRMR = 0.005). Internal consistency was also adequate for the three used scales (Composite reliability was 0.755 for the BRCS scale, while it ranged between a minimum of 0.855 and a maximum of 0.887 in the three subscales of the APRI and 0.853–0,850 for the “Psychological well-being” and “Mood and emotions” KIDSCREEN domains respectively.

### Resilience levels and HRQoL`s psycho-emotional domains according to sociodemographic characteristics

From a 0 to 100 range, the mean resilience levels of participants calculated through the BRCS questionnaire was 61.4 points (SD = 25.3 points). As shown in Table 2, no statistically significant results were found between adolescents’ resilience levels according to sociodemographic characteristics.

**Table 2:** Descriptive analyses of adolescents’ resilience according to sociodemographic characteristics.

	Resilience (BRCS) [0.0–100.0]
	Mean (CI 95%)
**Gender**	
Female	61.6 (59.7, 63.5)
Male	61.2 (59.3, 63.1)
**Age range (years)**	
[12.0–13.9]	62.6 (60.1, 65.1)
[14.0–15.9]	60.6 (58.6, 62.6)
[16.0–18.9]	61.4 (58.2, 64.6)
**Type of family**	
Nuclear	61.7 (60.3, 63.1)
Mononuclear	59.4 (55.5, 63.3)
No parents at home	58.8 (41.9, 75.7)
**Parental ethnic origin**^**2**^	
Both Spanish	61.7 (60, 63.4)
One Spanish	61.3 (52.8, 69.8)
Maghreb	58.1 (54.7, 61.5)
Latin-Ecuador	64.1 (59.7, 68.5)
Other	65.0 (59.2, 70.8)
**Social class**	
I-II	61.5 (58.7, 64.3)
III	62.6 (58.3, 66.9)
IV-V	62.1 (60.3, 63.9)
VI	57.2 (52.1, 62.3)
**Parental educational attainment**	
No education/primary education	61.2 (58.6, 63.8)
Secondary education	63.0 (61.1, 64.9)
Higher education	60.0 (57.0, 63.0)
**Total adolescent**	61.4 (36.1, 86.7)

*Null hypothesis* = *homogenous means (hypothesis contrast using the Oneway Analysis of Variance).*

*p < 0.05. Therefore, no statistically significant results were found.

a. Social class: I. Higher managerial; II. Intermediate managerial; III. Supervisory and junior managerial; IV. Skilled manual occupations; V. Unskilled manual occupations; VI. Unemployed/pensioner/retiree.

Results in Tables 3, 4 and 5 show how HRQoL’s levels decreased as the age of the adolescents increased. This specially occurred for “Moods and Emotions” domain, in particular when adjusted by social victimization (β =  − 1.22; CI 95% − 1.63, − 0.80).

**Table 3:** Associations between psychological domains of HRQoL and resilience among adolescents exposed to physical victimization.

	Psychological Well-being	Mood and Emotions
	β (CI 95%)	β (CI 95%)
Female vs Male	− .89 (− 1.88, 0.11)	− 2.15 (− 3.17, − 1.14)**
Age	− 0.95 (− 1.36, − 0.54)**	− 1.16 (− 1.58, − 0.73)**
Resilience	0.08 (0.06, 0.10)**	0.06 (0.04, 0.08)**
Physical victimization	− 0.14 (− 0.25, − 0.04)*	− 0.10 (− 0.21, − 0.01)*
**Interaction:** Physical victimization X Resilience	− 0.0005 (− 0.0023, 0.0013)	− 0.0021 (− 0.004, − 0.0003)*
R^2^	0.102	0.102
∆R^2^	0.002	0.004
*F Snedecor*	26.88**	26.17**

Associations between physical victimization and resilience on psychological domains calculated by linear regression analyses expressed through β coefficients and 95% confidence intervals (CI 95%).

*p < 0.05; **p < 0.001.

**Table 4:** Associations between psychological domains of HRQoL and resilience among adolescents exposed to social victimization.

	Psychological Well-being	Mood and Emotions
	β (CI 95%)	β (CI 95%)
Female vs Male	− 0.77 (− 1.75, 0.20)	− 2.00 (− 2.99, − 1.00)**
Age	− 0.99 (− 1.39, − 0.58)**	− 1.22 (− 1.63, − 0.80)**
Resilience	0.08 (0.06, 0.10)**	0.06 (0.04, 0.08)**
Social victimization	− 0.20 (− 0.30, − 0.10)**	− 0.15 (− 0.25, − 0.04)*
**Interaction:** Social victimization X Resilience	0.0003 (− 0.013, 0.0019)	− 0.0013 (-0.003, 0.0003)
R^2^	0.123	0.131
∆R^2^	0.0001	0.002
*F Snedecor*	33.25**	34.93**

Associations between social victimization and resilience on psychological domains calculated by linear regression analyses expressed through β coefficients and 95% confidence intervals (CI 95%).

*p < 0.05; **p < 0.001.

**Table 5:** Associations between psychological domains of HRQoL and resilience among adolescents exposed to verbal victimization.

	Psychological Well-being	Mood and Emotions
	β (CI 95%)	β (CI 95%)
Female vs Male	− 0.97 (− 1.96, 0.01)	− 2.21 (− 3.21, − 1.21)**
Age	− 0.98 (− 1.39, − 0.58)**	− 1.20 (− 1.62, − 0.79)**
Resilience	0.09 (0.07, 0.11)**	0.07 (0.04, 0.09)**
Verbal victimization	− 0.16 (− 0.26, − 0.06)**	− 0.10 (− 0.20, − 0.01)*
**Interaction:** Verbal victimization X Resilience	− 0.0002 (− 0.0018, 0.0013)	− 0.0018 (− 0.0034, − 0.0002)*
R^2^	0.121	0.125
∆R^2^	0.0001	0.004
*F Snedecor*	32.54**	33.01**

Associations between verbal victimization and resilience on psychological domains calculated by linear regression analyses expressed through β coefficients and 95% confidence intervals (CI 95%).

*p < 0.05; **p < 0.001.

A significant relation was also found between gender and HRQoL in “Moods and Emotions” dimension. Women obtained lower scores, with β-values ranging from a minimum of − 2.21 (CI 95% − 3.21, − 1.21) adjusted by verbal victimization to a maximum of − 2.00 (CI 95% − 2.99, − 1.00) adjusted by social victimization.

### Resilience as a moderator of relation between peer victimization and psycho-emotional domains of HRQoL

The main objective of this study was to examine adolescents’ resilience as a moderator of the relationship between physical, social and verbal victimization and the psychological domains of adolescents’ HRQoL.

It was observed how resilience levels had a directly proportional linear correlation with psychological well-being and mood levels, with β-values ranging from a minimum of 0.06 (CI 95%0.04, 0.08) adjusted by physical and social victimization to a maximum of 0.09 (CI 95% 0.07, 0.11) adjusted by verbal victimization (see Tables 3, 4 and 5).

Peer victimization was negatively associated with both domains of HRQoL. The negative association of violence was particularly stronger on psychological well-being, with social victimization reaching the highest β-values − 0.20 (CI 95% − 0.30, − 0.10).

The moderating effect of resilience between the association of peer victimization and HRQoL’s psycho-emotional dimensions was statistically significant between “Mood and Emotions” domain with physical victimization (β =  − 0.0021; CI 95% − 0.004, − 0.0003; *p* = 0.025) and verbal victimization (β =  − 0.0018; CI 95% − 0.0034, − 0.0002; *p* = 0.024), but not in case of social victimization. However, these relations were weak and accounted for only an additional 0.4% of variance in adolescents’ mood in both cases (∆R^2 ^ 0.004). However, no moderating effect on resilience was found between victimization in any of its forms and psychological well-being.

**Figure 2:**
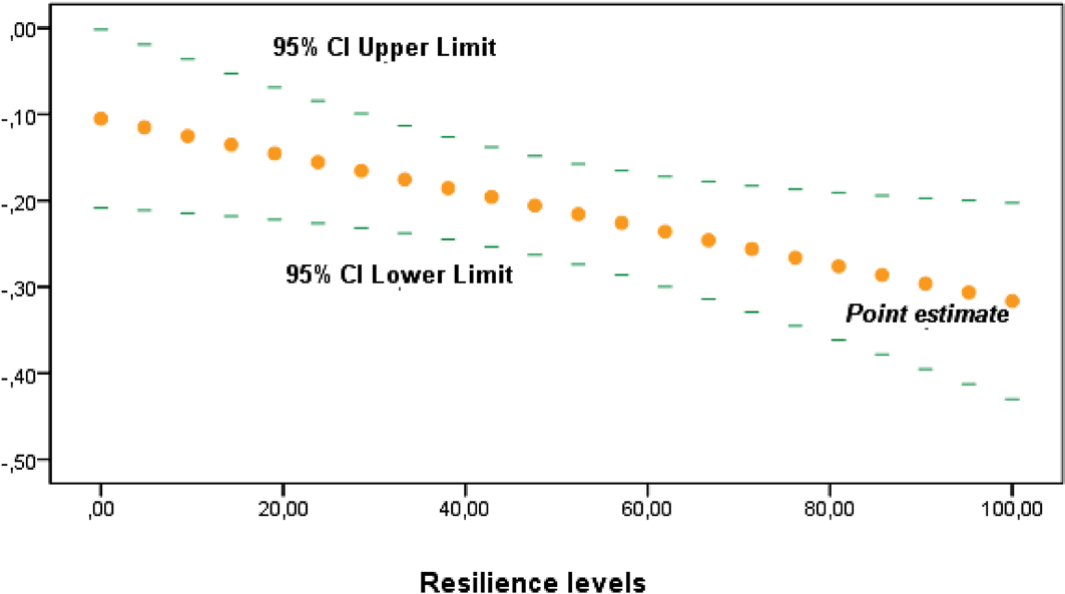
Johnson–Neyman method to study conditional effect of resilience as a moderator of the association between “Mood and Emotions” and Physical victimization. Y-axis represents β-coefficients.

**Figure 3:**
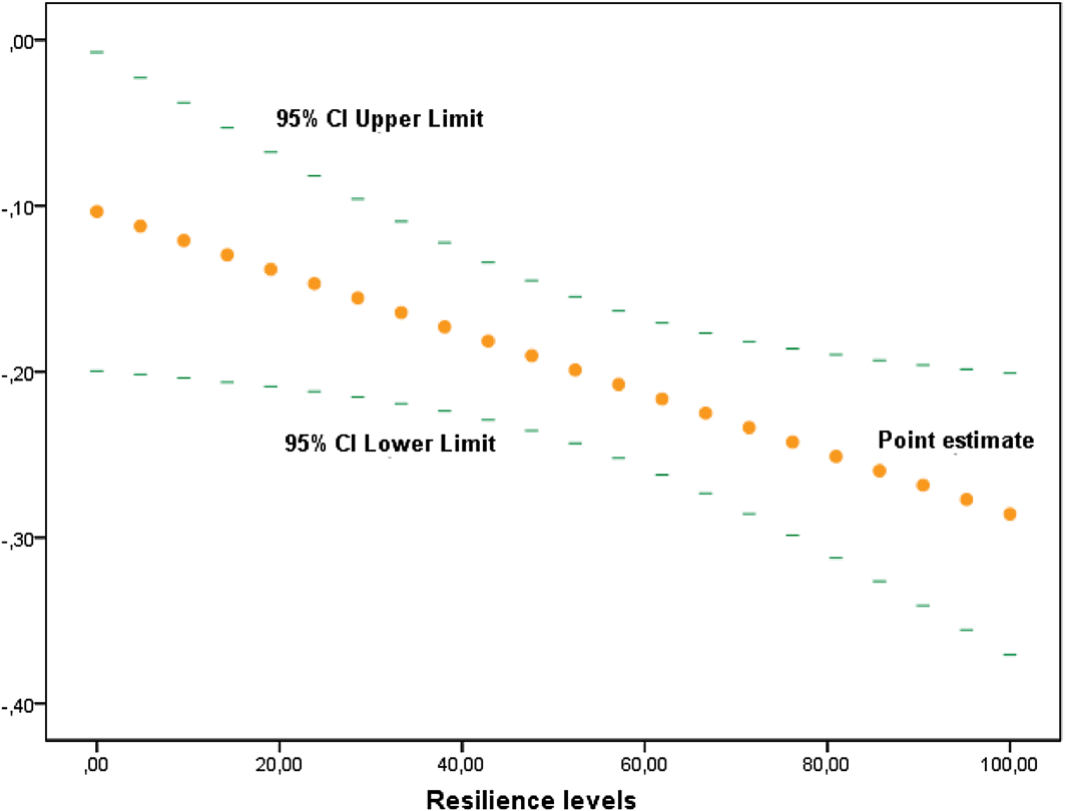
Johnson–Neyman method to study conditional effect of resilience as a moderator of the association between “Mood and Emotions” and Verbal victimization. Y-axis represents β-coefficients.

There were no statistical significance transition points within the moderating effect of resilience using the Johnson-Neyman method in any of the victimizations. (Figs. 2 and 3).

## Discussion

To our knowledge, this is one of the first studies to explore the association between specific types of peer victimization experienced by adolescents, resilience and its possible modulating power on HRQoL’s psychological dimensions. We sought to analyse whether adolescents’ resilience could function as a moderating factor of the relation between physical, social and verbal victimization and the HRQoL’s psychological domains.

This study suggests that resilience could regulate the association between physical and verbal victimization and the “Mood and Emotions” dimension of adolescents’ HRQoL. Nevertheless, this regulating influence appears to be faint since it only represents an additional 0.4% of variance in adolescents’ mood in both cases. On the other hand, no modulating effect of resilience was found related to victimization and HRQoL when it came to social victimization or the “Psychological Well-being” domain of HRQoL.

Although previous studies have considered resilience to be a powerful factor that mediated the relation between peer victimization and different adolescents’ health correlates^33^, other authors have also demonstrated that this regulating effect is weak^30,32^. The latter is the case of the present study, in which weak relations or none were found in the analysis of the associations between resilience, specific HRQoL’s dimensions and the most important types of peer victimization (physical, social and verbal).

According to prior literature^53,54^, the results of this research show that high resilience levels are associated with better psychological HRQoL. Likewise, those adolescents who reported having suffered peer victimization are those with the worst HRQoL in psychological terms^20^, obtaining the worst results in the case of psychological well-being when suffering social victimization. Therefore, as mentioned above, the results of the present study show that resilience is associated to peer victimization and psychological HRQoL, but no robust moderating effect of resilience was observed between peer victimization and psychological HRQoL. These results support theories proposed by other authors^30,31^: peer victimization may lead to a depletion or detriment of personal tools for coping with distress relationships or other adverse events, that is, it may lead to a depletion of resilience. In this sense, lower resilience levels in these adolescents mean that their modulatory capacity to avoid adverse effects on their HRQoL’s psychological sphere is reduced. This would explain the reduced regulatory power of resilience in these terms and, therefore, its association with lower levels of HRQoL in victims compared to not victimized adolescents. This hypothesis could lead us to suggest that social victimization negatively affects adolescents’ resilience capacity the most, since this study shows that this victimization is the only one in which resilience does not have any modulatory effect and, therefore, in which the worst levels of psychological HRQoL are observed.

In sum, these findings highlight the need of urgent effort into avoiding peer victimization in any of its forms, since all types of peer violence negatively impact adolescents’ HRQoL related to psychological domains. Previous research have shown that secondary schools are ideal settings for developing prevention programs aimed to reducing violence between peers ^55^, since they give the opportunity to interact directly with them in a practical way by role-playing, games, or any other activity that allows to work on avoiding violence in a stimulating way. On the other hand, results also suggest the importance of establishing surveillance programmes at schools to early detect teenagers who are experiencing violence. In this way, adolescent victims, whose personal strengths to manage disruptive relationships are exhausted, could be helped to strengthening their resources for better coping with undesirable life situations and, thus, minimize negative outcomes on their health. Previous studies have demonstrated strategies to reinforce personal strengths at secondary schools^56^.

The results of this study suggest that resilience could function as a protective factor that weakly regulates the negative association between physical and verbal victimization and the “Mood and Emotions” domain of adolescents’ HRQoL, however, we cannot know whether resilience has a stronger protective capacity on other components of the emotional well-being and health of victimized adolescents. For this reason, more research is needed to further investigate resilience trajectories in the context of peer victimization and its potential protective capacity over undesirable adolescents’ health outcomes.

Sociodemographic factors did not play a significant role between adolescents’ resilience levels in this research. Although other authors suggested that resilience may differ according to different cultural contexts^29,57^, this study is in line with those concluding that resilience is a process of adaptation^28^ and it is necessary to take into account previously accumulated adverse events to understand it^58^, being independent from the socio-demographic factors associated with an individual.

### Strengths and limitations

Potential limitations should be considered when interpreting the results. First, our findings must be interpreted in a specific socio-cultural context in which the investigation took place, so we cannot generalize the results to other regions. Although one third of the total participants were non-Spanish, further studies would be desirable to collect evidence from other regions or countries since results cannot be generalised to other populations. Second, it is not possible to make causal inferences because of the cross-sectional nature of the study. Future prospective investigations are recommended to clarify resilience behaviour in the context of peer victimization. Furthermore, due to the cross-sectional nature of this research levels of adolescents’ HRQoL could only be analysed at the time of data collection. Therefore, we can’t know how was their quality of life before being victimized. Third, the information was obtained from self-administered questionnaires so the possibility of recall bias cannot be rule out. Cross-checking adolescents' information by different sources would be desirable in future studies. Fourth, the quality of life construct has been partially measured, referring only to two of the KIDSCREEN dimensions (“Psychological Well-being” and “Mood and Emotions), which was considered to be the most relevant in our study. For this reason, we cannot affirm that the dependent variable was the “quality of life” during school age, but rather a partial aspect of it (the psychological components). However, according to the results of CFA these components of the quality of life seems to have its own entity by showing a good fit to the sample data. In addition, Internal consistency of both dimensions was also adequate. Finally, cyberbullying, a type of victimization that is becoming increasingly important, was not analysed. This may explain the lower prevalence of victimization in our study compared to others^7,8^ which have shown higher rates of this phenomenon. Future investigation should consider studying possible associations between resilience, cyberbullying and HRQoL’s psychological domains in order to compare outcomes with those from research that only focuses on traditional forms of peer victimization.

Notwithstanding these limitations, strengths of this study include the large sample size in conjunction with the significant participation rate (90.8%). The use of widely accepted questionnaires with scores that are valid and reliable in this study sample should also be taken into account. An interesting strength is also the separate analyses of the three most important types of peer victimization rather than the measurement of victimization as a whole.

## Conclusions

The results of this study suggest that resilience could function as a protective factor that weakly regulates the negative association between physical and verbal victimization and the “Mood and Emotions” domain of adolescents’ HRQoL. Therefore, future research directions should focus on exploring other protective factors that could stronger minimize the devastating consequences that peer victimization has on the psychological sphere of adolescents’ HRQoL. Thus, specific intervention programmes could be implemented to build and reinforce these strengths in victimized adolescents. Likewise, more research is needed to further investigate resilience trajectories in the context of peer victimization and its potential protective capacity over undesirable adolescents’ health outcomes.

## Supplementary Information

Supplementary Information 1.

Supplementary Information 2.

Supplementary Information 3.

## Data Availability

ll data generated or analysed during this study are included in this published article [and its supplementary information files].
