# Supplementary material for: The impact of resilience as a protective factor on Health-Related Quality of Life’s psychological dimensions among adolescents who experience peer victimization
Source: Sci Rep. 2022 Nov 7;12:18898. doi: 10.1038/s41598-022-23424-1 (PMC9640611; doi:10.1038/s41598-022-23424-1)
Supplement: Supplementary file 3 — Supplementary Information 3. [file 41598_2022_23424_MOESM3_ESM.docx]

**F1 (Gender):**

1 = MALE

2 = FEMALE

**EDAD_INT (age range):**

1 = 12.0–13.9 years

2 = 14.0–15.9 years

3 = 16.0–18.9 years

**F4_NOCONVIVEN_RR (Type of family):**

0 = Nuclear

1 = Mononuclear

2 = No parents at home

**F5_AMBOS (Parental ethnic origin):**

100 = Both Spanish

110 = One Spanish

300 = Maghreb

425 = Latin-Ecuator

800 = Other

**F6_MASALTA (Social class):**

1 = I/II

3 = III

5 = IV/V

6 = VI

**F7_MASALTA_R (Parental educational attainment):**

0 = No education / primary education

1 = Secondary education

2 = Higher education

**BRCS (Resilience levels)**

**KC52pw_T (52item Psychological Wellbeing international T-values based on RASCH PP)**

**KC52me_T (52item Mood and Emotions international T-values based on RASCH PP)**

**BULLYING_FIS (Physical victimization)**

**BULLYING_SOC (Social victimization)**

**BULLYING_VERB (Verbal victimization)**

**BULLYING_TOT (Total victimization)**

**BRCSXBULLYING_TOT (Interaction between resilience and total victimization)**

**BRCSXBULLYING_FIS (Interaction between resilience and physical victimization)**

**BRCSXBULLYING_VERB (Interaction between resilience and verbal victimization)**

**BRCSXBULLYING_SOC (Interaction between resilience and social victimization)**
